# Serum sclerostin in high-activity adult patients with juvenile idiopathic arthritis

**DOI:** 10.1186/s13075-014-0460-x

**Published:** 2014-10-04

**Authors:** Kristyna Brabnikova-Maresova, Katerina Jarosova, Karel Pavelka, Jan J Stepan

**Affiliations:** Institute of Rheumatology, Na Slupi 4, 128 50 Prague 2, Czech Republic; Department of Rheumatology, First Faculty of Medicine, Charles University in Prague, Kateřinská 32, 121 08 Prague 2, Czech Republic

## Abstract

**Introduction:**

Juvenile idiopathic arthritis (JIA) is a disease associated with loss of bone mass, deterioration in bone mass quality and an increased risk of fractures. The objective of this study was to evaluate factors that predict bone mineral density (BMD) alterations in young adult patients with active JIA before and during therapy with tumour necrosis factor α (TNFα) inhibitors.

**Methods:**

Thirty-one patients (twelve males and nineteen females; mean age =25.1 ± 6.1 years) with active JIA (mean Disease Activity Score in 28 joints (DAS28) =6.36 ± 0.64; mean high-sensitivity C-reactive protein (hsCRP) =18.36 ± 16.95 mg/L) were investigated. The control group consisted of 84 healthy individuals matched by sex and age. BMD, bone turnover markers and serum concentrations of soluble receptor activator of nuclear factor κB ligand, osteoprotegerin, dickkopf Wnt signalling pathway inhibitor 1 (Dkk1) and sclerostin were evaluated.

**Results:**

Baseline BMD values in the lumbar spine, proximal femur, femoral neck and distal radius were significantly lower in patients with JIA compared to healthy control participants. Baseline sclerostin serum concentrations were significantly higher in patients with JIA compared to control participants. After 2 years of treatment with TNFα inhibitors, BMD was significantly increased in the lumbar spine. This increase correlated with a drop in DAS28 score. A statistically significant correlation between hsCRP and Dkk1 was found at baseline, as well as during the 2-year follow-up period. A significant reduction in serum sclerostin after 1 year of therapy was predictive of a drop in DAS28 score observed with a 1-year delay after reduction of serum sclerostin.

**Conclusion:**

A significant correlation between the sclerostin serum concentration and the number of tender and swollen joints, but not BMD, supports the hypothesis that chondrocytes and cells of the subchondral bone may contribute to circulating sclerostin in JIA.

## Introduction

Juvenile idiopathic arthritis (JIA) is a systemic autoimmune inflammatory connective tissue disease with onset occurring before age 16 years. It is associated with a decrease in bone mass, thinning of the cortical bone [1,2], sarcopenia [3,4] and an increased risk of fractures [5]. Bone loss may be systemic or localized to the periarticular bone due to arthritis of the affected joint. The pathophysiology of the bone mass loss may involve, in particular, the negative effect of proinflammatory cytokines, as well as treatment with glucocorticoids [6]. Thus, the loss of bone mass may reflect a disorder of bone modelling and remodelling. This process involves proinflammatory cytokines produced by the synovial membrane, which may increase bone resorption but also stimulate soluble antagonists of the canonical Wnt/β-catenin signalling pathway, including dickkopf Wnt signalling pathway inhibitor 1 (Dkk1) and sclerostin, and subsequently inhibit osteoblast proliferation, maturation and progenitor differentiation [7-20]. The significance of Wingless (Wnt) proteins in susceptibility to JIA was confirmed in a study of polymorphisms in the Wnt-1-inducible signalling pathway protein 3 (WISP3) [21]. To the best of our knowledge, no study related to circulating sclerostin or Dkk1 in patients with JIA has been published to date.

In the present study, we assessed bone mineral density (BMD) at standard skeletal sites, as well as biochemical markers of osteoclast, osteoblast and osteocyte function, in patients with persistent high-activity JIA. These parameters were assessed at baseline and after 12 months and 24 months of treatment with tumour necrosis factor α (TNFα) inhibitors. Young adults with JIA were included in the study. The objective of the study was to evaluate factors that predict BMD alterations in young adult patients with active JIA being treated with TNFα inhibitors.

## Methods

### Study population

We conducted a prospective, open-label study in 2009 and 2010. We enrolled 31 patients with JIA (12 males and 19 females) with a mean age of 25.1 ± 6.1 years who had high disease activity determined on the basis of high-sensitivity C-reactive protein (hsCRP) level, erythrocyte sedimentation rate (ESR) and Disease Activity Score in 28 joints (DAS28). All of the included patients met the criteria of the Czech Rheumatology Society for treatment with TNFα inhibitors. At baseline, the patients were naive to anti-TNFα therapy. The basic condition for inclusion in the study was high disease activity expressed by DAS28 ≥ 3.9. Another precondition was inadequate response to one disease-modifying antirheumatic drug [22].

At the time of patient selection, a group of healthy control participants was recruited from amongst the friends, acquaintances and colleagues of the patients with JIA. In total, 100 healthy males and females were examined. Each patient with JIA was matched with three suitable control participants on the basis of sex and age (in most cases within a 2-year age difference and in isolated cases within a maximum age difference of 6 years). Nine female patients with JIA were matched with two control participants only. In this manner, 84 age- and sex-matched control participants were selected. All study participants were examined and treated at the Institute of Rheumatology in Prague.

The study was conducted in accordance with the World Medical Association Declaration of Helsinki regarding ethical conduct of research involving human subjects. The study was approved by the ethics committee of the Institute of Rheumatology, and all participants gave us their written informed consent before enrolment.

### Clinical parameters

The baseline clinical status of the patients with JIA was established on the first day of TNFα inhibitor treatment. A physical examination, blood testing and BMD testing were performed on day 1 and again at 12 and 24 months. Detailed information about each patient’s JIA disease course and treatment, as well as personal history, family history and fracture risk factor assessment, was obtained from the patient and the available medical documentation.

A physical examination of the patients with JIA was performed at each visit, and the number of swollen and tender joints was documented. Body height was measured using a stadiometer. Body weight was measured using an exact scale. The control participants were examined in a similar manner.

The patients with JIA were given daily supplements of 1,000 mg of calcium and 800 IU of vitamin D for a minimum of 6 months before the start of anti-TNFα treatment. The control participants did not receive any calcium or vitamin D supplements.

To maintain the same conditions for the collection of blood samples, patients and control participants were instructed to fast for 12 hours overnight while maintaining adequate hydration. At 8:00 the following morning, venous blood was collected into regular laboratory serum tubes by antecubital venepuncture. For the purposes of soluble receptor activator of nuclear factor κB ligand (sRANKL), osteoprotegerin (OPG), Dkk1 and sclerostin assays, aliquots of blood serum were frozen and stored at −80°C until analysis after the study was completed. Other laboratory parameters were measured on the day of blood collection.

For the purpose of determining JIA activity, the DAS28 score was calculated using objective clinical components (the number of swollen and tender joints among the total of 28 joints assessed), laboratory components (ESR in millimetres per hour) and subjective components (patient global health analogue scale, mm) (PGH).

### Laboratory methods

The hsCRP concentration was measured by immunoturbidimetry. The interassay coefficient of variation was 1.9%.

The sRANKL, OPG, Dkk1 and sclerostin serum concentrations were measured using enzyme-linked immunosorbent assay (ELISA) kits in compliance with the manufacturer’s instructions, always by the same person. The results were read using the Tecan Sunrise ELISA reader (Tecan, Männedorf, Switzerland). All samples were analysed in duplicates. The sRANKL ELISA (Biomedica Medizinprodukte, Vienna, Austria) was performed to detect free sRANKL without the OPG bond; intra- and interassay coefficients of variation were 9% and 3%, respectively; and the limit of detection was 0.02 pmol/L. The serum OPG ELISA (Biomedica Medizinprodukte) intra- and interassay coefficients of variation were 10% and 7%, respectively, and the limit of detection was 0.14 pmol/L. The Dkk1 ELISA (Biomedica Medizinprodukte) intra- and interassay coefficients of variation were 7% and 8%, respectively, and the limit of detection was 0.38 pmol/L. The serum sclerostin ELISA kit (Uscn Life Science, Wuhan, China/Cloud-Clone, Houston, TX, USA) has no significant cross-reactivity or interference between human sclerostin and analogues; the standard curve is read between 0.312 and 20 g/L. The limit of detection was 0.106 g/L, and the intra- and interassay coefficients of variation were <10% and <12%, respectively.

The bone metabolism markers osteocalcin (OC), procollagen type I N-terminal propeptide (PINP), C-terminal telopeptide of collagen type I (βCTX-I) and 25-hydroxyvitamin D_3_ were measured using electrochemiluminescence-based immunoanalysis (cobas analyzer; Roche Diagnostics, Mannheim, Germany). The intraassay coefficient of variation for OC was <5% at concentrations between 11 and 40 μg/L. The intraassay coefficient of variation for PINP was <5%, and the interassay coefficient of variation was <7%, at concentrations between 20 and 90 μg/L. The intraassay coefficient of variation for βCTX-I was <7% for samples between 200 and 500 ng/L and <10% for very low βCTX-I concentration samples. The interassay coefficient of variation for βCTX-I was <9% for samples between 200 and 500 ng/L. The detection limit for βCTX-I was <10 ng/L.

### Imaging parameters

Radiographs of the thoracic and lumbar spine (anteroposterior and lateral) were obtained. BMDs of the lumbar spine, proximal hip, femoral neck and distal forearm were measured by dual-energy X-ray absorptiometry using a bone densitometer (Prodigy; GE Healthcare, Waukesha, WI, USA). The same technician performed all measurements using the same instrument, and the same physician performed all assessments. The short-term *in vivo* coefficients of variation for lumbar spine, total femur, femoral neck and distal radius were 0.7%, 0.9%, 1.8% and 0.9%, respectively. The long-term coefficient of variation using the Hologic phantom was 0.31%. Daily scanning of a phantom showed an absence of machine drift during the study. BMD was expressed in grams per square centimetre.

### Statistical analysis

Data for continuous variables were expressed as the mean ± SD or the median with a 75% confidence interval (CI). Bivariate analyses were performed by using Student’s *t*-test and Pearson’s correlation coefficients to analyse the relationships between the dependent variables. Following anti-TNFα treatment, changes in the monitored parameters (BMD at individual skeletal locations at 12 and 24 months, laboratory parameters at 6, 12 and 24 months) were compared using one-way repeated-measures analysis of variance (ANOVA). These changes in the JIA group were compared with those in the control group by using one-way ANOVA. A stepwise multiple linear regression analysis was performed to create a multivariate summary model of the determinants of dependent variables. All assumptions of the linear regression analysis were also examined. Results at the level of *P* =0.05 were considered significant. All analyses were performed using a SigmaPlot 10 software package (Systat Software, Erkrath, Germany).

## Results

The basic clinical and laboratory characteristics of the participants are listed in Tables 1, 2 and 3. In female patients with JIA, the average age of menarche was 13.1 ± 1.2 years. No significant differences in anthropometric parameters were observed between the control participants and patients with JIA. In males and females with JIA, significant sex-based differences were identified in body height, DAS28 score, OC, PINP, βCTX-I and BMD in the distal radius (Table 2). In patients with JIA, the disease had been diagnosed at approximately age 10.3 ± 4.8 years, and it had lasted on average for 14.6 ± 9.1 years at the time of the study. The cervical spine was affected in seven patients. None of the patients in the study had vasculitis or lung impairment. Six patients tested positive for antinuclear antibodies. The polyarticular form of JIA was present in sixteen patients. Four patients were rheumatoid factor–positive, and twelve were rheumatoid factor–negative. The enthesitis-related form of JIA was present in nine patients; the extended oligoarticular form was present in four patients; and the psoriatic form was present in two patients. Eighteen patients were in functional class I, four were in functional class II, five were in functional class III and four were in functional class IV. The mean Health Assessment Questionnaire score (1978) was 0.97 ± 0.60, and the mean EuroQol questionnaire score 5D was 0.56 ± 0.27. Before the start of anti-TNFα treatment, all of the patients with JIA were treated with disease-modifying antirheumatic drugs. Twenty-three patients were given methotrexate at an average dose of 16.8 ± 3.4 mg/wk; four patients were treated with leflunomide, two patients were treated with sulphasalazine; one patient was treated with sulphasalazine and hydroxychloroquine sulphate; and one patient was treated with cyclosporine A. Twelve patients were receiving glucocorticoid therapy (prednisone in ten patients and methylprednisolone in two patients). The average daily dose of glucocorticoids was 6.6 ± 4.3 mg; the median dose was 5 mg/day; and the dose range was 4 to 20 mg/day. Fourteen of the patients with JIA had taken glucocorticoids in the past, but did not during the course of this study. Five of the patients with JIA did not take glucocorticoids at any time.

**Table 1:** **Anthropometric and clinical characteristics of patients with juvenile idiopathic arthritis and control participants** ^**a**^

	**Patients with JIA** **(** ***n*** **=31)**	**Control participants** **(** ***n*** **=84)**	***P*** **-value**
Males/females (*n*)	12/19	36/48	0.692
Age (yr)	25.1 ± 6.1	23.8 ± 4.5	0.405
Height (cm)	170.5 ± 9.8	173.3 ± 9.3	0.145
Weight (kg)	68.0 ± 12.5	69.2 ± 12.5	0.884
BMI (kg/m^2^)	23.4 ± 3.9	22.9 ± 3.0	0.435
Vertebral fractures (*n*)	5	0	<0.001
Nonvertebral fractures (*n*)	6	0	<0.001
Family hip fracture history (*n*)	0	0	
Smoking (*n*)	7	13	0.372
Alcohol abuse (*n*)	0	0	
Glucocorticoids (*n*)	12	0	<0.001
Menarche (yr)	13.1 ± 1.2	12.9 ± 1.1	0.994
Contraception in females (*n*)	11	23	0.462
25(OH)D (nmol/L)	54.3	2.4	0.029
	(20.2 to 168.7)	(30.7 to 64.9)	
PTH (pmol/L)	4.8	5.9	0.004
	(1.3 to 8.8)	(4.7 to 7.6)	
Oestradiol (pmol/L)	98.7	103.2	0.438
	(18.4 to 541.1)	(56.4 to 260.3)	

^a^Displayed are numbers or means ± SD and/or medians and 75% CIs. Characteristics with zero value were not statistically calculated. BMI: Body mass index; JIA: Juvenile idiopathic arthritis; 25(OH)D: 25-hydroxyvitamin D_3_; PTH: Parathyroid hormone.

**Table 2:** **Clinical characteristics of patients with juvenile idiopathic arthritis by sex** ^**a**^

	**Men with JIA** **(** ***n*** **=12)**	**Women with JIA** **(** ***n*** **=19)**	***P*** **-value**
Age (yr)	21	26	0.079
	(19.75 to 26.5)	(20.5 to 32.8)	
Height (cm)	178.8 ± 7.6	165.2 ± 7.0	<0.001
Weight (kg)	73.3 ± 11.7	64.6 ± 12.1	0.059
BMI (kg/m^2^)	22.9 ± 2.9	23.7 ± 4.5	0.559
Disease duration (yr)	11.0 ± 8.2	16.9 ± 9.4	0.087
Vertebral fractures (*n*)	3	2	
Nonvertebral fractures (*n*)	2	4	
Smoking (*n*)	3	4	
Glucocorticoids (*n*)	3	9	0.230
Menarche (yr)		13.1 ± 1.2	
Contraception (*n*)		11	
hsCRP (mg/L)	9.65	13.78	0.395
	(5.84 to 44.78)	(6.78 to 24.21)	
DAS28	6.07	6.44	0.041
	(5.63 to 6.52)	(6.15 to 7.04)	
ESR (mm/hr)	24.5	26.0	0.670
	(17.0 to 54.0)	(24.0 to 34.5)	
Swollen joints (*n*)	11.0	12.0	0.263
	(7.5 to 13.5)	(9.0 to 18.3)	
Tender joints (*n*)	13.5	17.0	0.038
	(10.0 to 15.0)	(12.3 to 21.5)	
PGH (mm)	67.5	69.0	0.947
	(60.0 to 74.5)	(53.8 to 77.5)	
Osteocalcin (μg/L)	24.3	14.3	0.016
	(20.2 to 28.0)	(11.3 to 23.0)	
PINP (μg/L)	57.6	40.1	0.003
	(46.3 to 85.6)	(19.9 to 46.4)	
βCTX-I (μg/L)	0.54	0.23	<0.001
	(0.43 to 0.87)	(0.14 to 0.36)	
PTH (pmol/L)	4.08	4.80	<0.001
	(1.73 to 6.05)	(2.50 to 6.15)	
Lumbar spine BMD (g/cm^2^)	1.018	1.075	0.392
	(0.913 to 1.201)	(1.012 to 1.221)	
Total femur BMD (g/cm^2^)	0.938	0.877	0.073
	(0.887 to 1.131)	(0.764 to 0.916)	
Femoral neck BMD (g/cm^2^)	0.934	0.875	0.092
	(0.853 to 1.132)	(0.805 to 0.948)	
Distal radius BMD (g/cm^2^)	0.751	0.667	0.030
	(0.677 to 0.792)	(0.606 to 0.704)	

^a^Displayed are numbers or means ± SD and/or medians and 75% CIs. BMD: Bone mineral density; BMI: Body mass index; βCTX-I: C-terminal telopeptide of collagen type I; DAS28: Disease Activity Score in 28 joints; ESR: Erythrocyte sedimentation rate; hsCRP: High-sensitivity C-reactive protein; JIA: Juvenile idiopathic arthritis; 25(OH)D: 25-hydroxyvitamin D_3_; PINP: Procollagen type I N-terminal propeptide; PTH: Parathyroid hormone; PGH: patient global health.

**Table 3:** **Disease activity, bone mineral density and laboratory variables in patients with juvenile idiopathic arthritis during tumour necrosis factor α blocker treatment and in control participants** ^**a**^

	**0** **(** ***n*** **=31)**	**12 months** **(** ***n*** **=31)**	**24 months** **(** ***n*** **=31)**	**Control participants** **(** ***n*** **=83)**
hsCRP (mg/L)	12.09^b^	5.19^b^	4.28^b^	0.73
	(6.28 to 25.17)	(1.30 to 12.89)	(0.89 to 13.44)	(0.27 to 1.78)
ESR (mm/hr)	25.50^b^	16.00^b^	12.00^b,c^	4.00
	(20.00 to 36.00)	(4.00 to 24.00)	(4.00 to 26.00)	(2.00 to 8.00)
DAS28	6.26	2.93^c^	2.51^c^	
	(5.87 to 6.82)	(1.13 to 3.69)	(1.25 to 3.64)	
Swollen joints (*n*)	12.0	0.0^c^	0.0^c^	
	(9.0 to 15.5)	(0.0 to 2.0)	(0.0 to 2.0)	
Tender joints (*n*)	15.0	1.0^c^	0.0^c^	
	(11.3 to 17.8)	(0.0 to 2.0)	(0.0 to 2.0)	
PGH (mm)	69.0	18.0^c^	10.0^c^	
	(59.0 to 75.0)	(5.0 to 27.8)	(0 to 20.0)	
Lumbar spine BMD (g/cm^2^)	1.065^b^	1.089^b,c^	1.093^b,c^	1.209
	(0.972 to 1.221)	(1.044 to 1.248)	(1.053 to 1.161)	(1.175 to 1.279)
Femur total BMD (g/cm^2^)	0.909^b^	0.918^b^	0.886^b^	1.134
	(0.776 to 0.984)	(0.776 to 1.015)	(0.790 to 0.996)	(1.061 to 1.202)
Femoral neck BMD (g/cm^2^)	0.899^b^	0.903^b^	0.905^b^	1.118
	(0.819 to 1.017)	(0.806 to 1.033)	(0.821 to 1.019)	(1.042 to 1.201)
Distal forearm BMD (g/cm^2^)	0.690	0.712	0.707	0.713
	(0.614 to 0.761)	(0.638 to 0.769)	(0.632 to 0.762)	(0.676 to 0.768)
Osteocalcin (μg/L)	17.75	17.45	15.20	21.20
	(12.40 to 24.80)	(8.80 to 26.90)	(11.95 to 20.20)	(16.68 to 26.90)
PINP (μg/L)	46.16	46.41	51.23	50.96
	(31.96 to 59.41)	(32.01 to 88.77)	(33.66 to 72.01)	(37.10 to 81.84)
βCTX-I (μg/L)	0.35	0.33	0.33^b^	0.46
	(0.18 to 0.52)	(0.23 to 0.49)	(0.20 to 0.45)	(0.34 to 0.70)
sRANKL (pmol/L)	0.08	0.05	0.07	0.06
	(0.02 to 0.25)	(0.00 to 0.25)	(0.00 to 0.21)	(0.00 to 0.16)
OPG (pmol/L)	3.84	3.26	3.02	3.14
	(2.53 to 4.84)	(2.10 to 3.64)	(2.03 to 3.55)	(2.62 to 3.85)
sRANKL/OPG ratio	0.03	0.03	0.01	0.02
	(0.01 to 0.07)	(0.00 to 0.10)	(0.00 to 0.10)	(0.00 to 0.05)
Dkk1 (pmol/L)	28.74	24.08	21.52^b,c^	28.74
	(23.8 to 36.84)	(14.54 to 29.41)	(15.72 to 31.25)	(23.80 to 36.84)
Sclerostin (μg/L)	7.42^b^	2.06^c^	1.22^c^	1.94
	(3.57 to 15.49)	(1.00 to 3.48)	(0.30 to 3.26)	(0.62 to 5.71)

^a^BMD: Bone mineral density; BMI: Body mass index; βCTX-I: C-terminal telopeptide of collagen type I; DAS28: Disease Activity Score in 28 joints; PGH: patient global health; Dkk1: Dickkopf Wnt signalling pathway inhibitor 1; ESR: Erythrocyte sedimentation rate; hsCRP: High-sensitivity C-reactive protein; JIA: Juvenile idiopathic arthritis; 25(OH)D: 25-hydroxyvitamin D_3_; OPG: Osteoprotegerin; PINP: Procollagen type I N-terminal propeptide; PTH: Parathyroid hormone; sRANKL: Soluble receptor activator of nuclear factor κB ligand. ^b^Median and 75% CI with probability *P* <0.05 by means of one-way analysis of variance, as compared to control participants. ^c^Median and 75% CI with probability *P* <0.05 by means of repeated-measures one-way analysis of variance within JIA group, as compared to baseline.

At baseline, the BMD values (g/cm^2^) in all of the measured skeletal locations in patients with JIA were significantly lower compared to the healthy control participants (Table 3). A significant negative correlation between BMD in the femoral neck and the use of glucocorticoids (*r* = −0.40, *P* <0.05) and the disease duration (*r* = −0.48, *P* <0.005) was established for the baseline values. There was a statistically significant positive correlation between Dkk1 (*r* =0.36, *P* <0.05) and disease activity expressed by means of hsCRP. Following adjustment for sex, glucocorticoid treatment and disease duration, there was a significant correlation between DAS28 values and levels of OC (*P* =0.005) and OPG (*P* =0.32), but not βCTX-I. The baseline βCTX-I values were dependent on sex only (*P* <0.001). No significant association between sclerostin serum concentrations and other monitored variables was found, except for a correlation between serum sclerostin and oestradiol concentrations, following adjustment for sex (*P* =0.022).

In the course of the study, 18 patients were treated with infliximab, 8 patients were treated with etanercept and 5 patients were treated with adalimumab. The values of individual variables are provided in Table 3. The correlations among variables measured at baseline and in the course of treatment are given in Table 4.

**Table 4:** **Pearson correlation coefficients for the variables measured at baseline and during tumour necrosis factor α inhibitor treatment** ^**a**^

	**GCs**	**Duration**	**Lumbar spine BMD**	**Femur neck BMD**	**Dkk1**	**Sclerostin**	**hsCRP**	**DAS28**	**ESR**	**Tender joint counts**
Sex	0.226^b^	0.311^c^	0.074	0.350^c^	0.281^c^	−0.029	−0.124	0.280	0.085^c^	0.211^b^
GCs		0.130	0.274^c^	0.391^d^	0.291^c^	0.229^b^	0.229^b^	0.416^d^	0.330^c^	0.323^c^
Duration			0.176	−0.500^d^	−0.021	0.010	0.100	0.192	0.100	0.104
Lumbar spine BMD				0.512^d^	−0.106	−0.178	−0.055	−0.121	−0.075	−0.071
Femoral neck BMD					0.017	−0.154	−0.113	−0.270^b^	−0.266^b^	−0.116
Dkk1						0.026	0.402^d^	0.113	0.244	0.071
Sclerostin							−0.011	0.317^c^	0.023	0.332^c^
hsCRP								0.318^c^	0.751^d^	0.089
DAS28									0.594^d^	0.857^d^
ESR										0.255^b^

^a^BMD: Bone mineral density; DAS28: Disease Activity Score in 28 joints; Dkk1: Dickkopf Wnt signalling pathway inhibitor 1; ESR: Erythrocyte sedimentation rate; GCs: Glucocorticoids; hsCRP: High-sensitivity C-reactive protein. ^b^ *P* <0.05; ^c^ *P* <0.005; ^d^ *P* <0.001.

**Figure 1:**
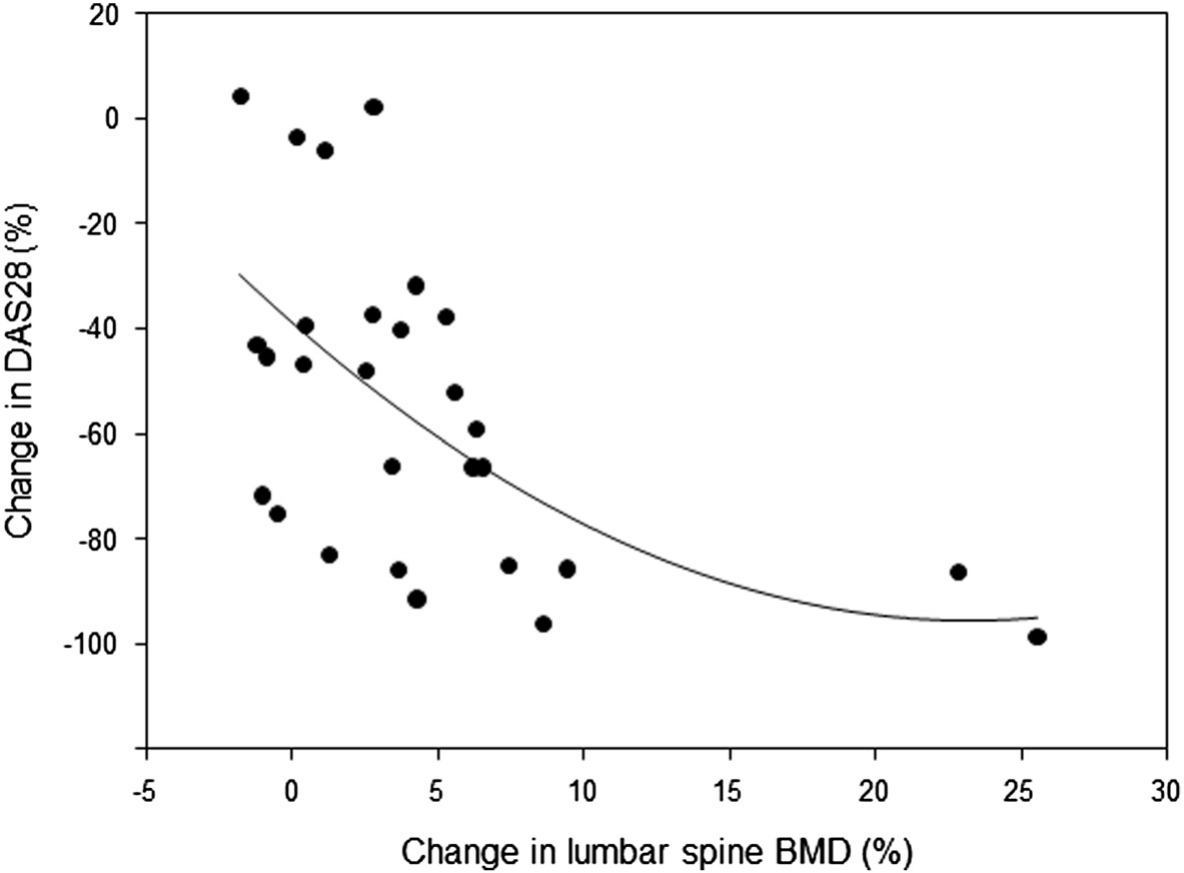
**Correlation between percent change in Disease Activity Score in 28 joints after 1 year and change in lumbar spine bone mineral density after 2 years of tumour necrosis factor α inhibitor treatment.** Change in bone mineral density (BMD) after 24 months = −1.582 − (0.112 × ∆DAS28 after 12 months) (*r* =0.53, *P* =0.003). DAS28: Disease Activity Score in 28 joints.

The values of BMD (g/cm^2^) increased in all of the measured skeletal locations compared to baseline values after the first year and after the second year of TNFα inhibitor treatment, respectively, by 2.9 ± 4.7% and by 4.6 ± 6.3% in the lumbar spine, by 0.8 ± 3.2% and by 1.0 ± 4.0% in the total proximal femur, and by 0.3 ± 3.9% and by 1.1 ± 5.0% in the femoral neck. However, the BMD increase was significant in the lumbar spine only (Table 3). An increase in spinal BMD after 2 years was significantly predicted by a reduction in DAS28 score after the first year of treatment (*P* =0.003), and this correlation did not change after adjustment for sex, age, body height, treatment with glucocorticoids or disease duration (Figure 1). Even after 2 years of TNFα inhibitor treatment, however, the lumbar spine BMD remained lower than that in the control group (Table 3).

**Figure 2:**
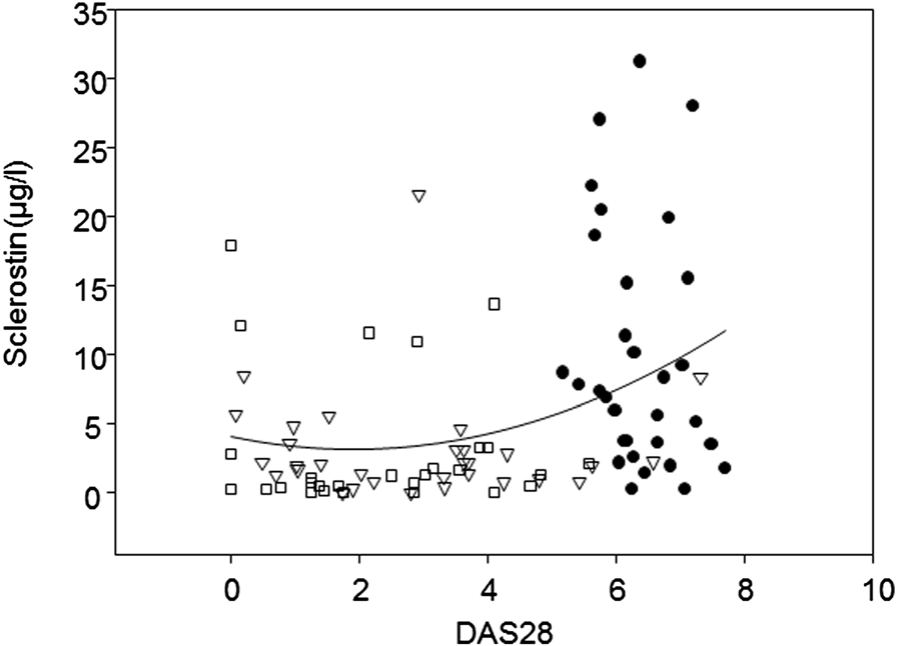
**Correlation between the values of Disease Activity Score in 28 joints and serum sclerostin concentration before treatment and during tumour necrosis factor α inhibitor treatment.** Disease Activity Score in 28 joints (DAS28) =3.332 + (0.104 × sclerostin) (*r* =0.32, *P* =0.002). Filled circles: baseline values; open triangles: 1 year of treatment; open squares: 2 years of treatment.

Before the start of anti-TNFα treatment, serum sclerostin concentrations were significantly higher than those in the control group. Serum sclerostin dropped significantly after as little 1 year of treatment (Table 3). Serum sclerostin concentrations did not correlate with ESR or hsCRP levels (Table 4). A significant correlation was found between the number of tender joints and serum sclerostin concentration after adjustment for ESR, sex and glucocorticoid therapy (*r* =0.40, *P* <0.001). The correlation between the values of DAS28 and serum sclerostin concentration before treatment and during anti-TNFα treatment (Figure 2, Table 4) remained significant after adjustment for treatment with glucocorticoids (*r* =0.37, *P* =0.002). There was a significant positive correlation between hsCRP levels (after adjustment for treatment with glucocorticoids) and Dkk1 serum concentrations (*r* =0.40, *P* <0.001) after 2 years of anti-TNFα treatment.

A significant negative correlation was observed between serum βCTX-I concentrations and BMD at the total proximal femur (*r* = −0.44, *P* <0.001) and the femoral neck (*r* = −0.41, *P* <0.001). These correlations lost their significance after adjustment for age, sex, body height, disease duration and treatment with glucocorticoids, however.

## Discussion

Testing of patients with JIA with high disease activity confirmed that they had significantly lower BMD in all of the measured skeletal locations compared to the control group. BMD in the proximal femur depended upon disease duration and treatment with glucocorticoids [23,24]. During anti-TNFα treatment, BMD increased. A significant increase in the lumbar spine BMD after 2 years of treatment correlated with the drop in DAS28 values [25,26]. However, BMD changes should be interpreted with caution, as we did not follow up BMD in control participants, and it is unclear whether peak BMD was already achieved in all patients. Furthermore, hip BMD remained unchanged and lower than in control participants.

At baseline, there was a significant positive correlation between DAS28 values and the serum concentrations of sclerostin, as well as with hsCRP, but no significant association between the values of hsCRP and sclerostin was established in this study. The association between baseline values of DAS28 and serum concentrations of OC, but not βCTX-I, suggests the inhibition of new bone mass formation in active inflammatory JIA. This is consistent with the finding that etanercept is able to activate osteoblastogenesis and new bone formation by means of Dkk1 inhibition in the rheumatoid arthritis (RA) rat model [27].

Sclerostin serum concentrations depend on genetic aspects, as well as age, sex, adiposity, kidney function and presence of diabetes mellitus [28]. In the present study, the use of an age-homogeneous group of young individuals obviated effect of age on sclerostin levels [29,30]. Although the treated patients with JIA exhibited significantly higher sclerostin serum concentrations than the control participants, no significant association between sclerostin serum concentration and any other monitored variable (for example, BMD) was found at baseline. In an arthritis mouse model, sclerostin inhibition resulted in a decrease in the loss of bone mass [31,32]. It is necessary, however, to point out that the values of sclerostin serum concentrations depend on the type of immunoanalysis used for measurement [33]. The literature reveals contradictory results concerning the association between sclerostin serum concentrations and BMD of the femoral neck in postmenopausal women [29,30,34,35]. The association between serum sclerostin and the risk of fractures was established in two studies in postmenopausal women [36,37], but was not evidenced in another study [34].

Significant reduction in the expression of sclerostin occurs during mechanical loading of the skeleton [38,39]. Sclerostin is not a specific product of osteocytes, however [40]. Sclerostin is also produced by chondrocytes and cementocytes [38,41], as well as in the liver, vascular wall and kidney [42,43]. Our present study in young adults with active JIA provides evidence of an association between sclerostin serum concentrations and the disease activity assessed by DAS28 values, but not by hsCRP values. Although sclerostin and Dkk1 represent relevant inhibitors of the Wnt signalling pathway and subsequently of new bone mass formation by osteoblasts [8,44-46], causal dependence between the known biological effect of sclerostin (inhibition of new bone formation) and BMD was not evidenced in our patients. Both subchondral bone cells and chondrocytes may be sources of circulating sclerostin patients with JIA. This hypothesis is supported by the finding that, in the RA model, the sclerostin inhibition by a monoclonal antibody protects the bone and cartilage from inflammatory damage [31]. This hypothesis is further supported by a significant correlation between serum sclerostin and the number of tender and swollen joints, as established in our study, whereas no significant correlation between serum sclerostin and hsCRP, ESR or Dkk1 has been identified.

Although baseline serum Dkk1 levels were not increased in patients with JIA compared to healthy control participants, there was a significant positive correlation between Dkk1 levels and disease activity expressed by hsCRP. The relationship between hsCRP and Dkk1 values was confirmed by a significant drop in both of these markers after 2 years of treatment. A similar dependence between hsCRP and Dkk1, as well as a drop in both variables, was previously described in patients with RA treated with infliximab [20]. The patients with JIA in our study did not exhibit any significant relationship between the Dkk1 serum concentrations and any of the other monitored variables at baseline or during treatment with TNFα inhibitors.

In this study, we did not find any significant increase in the serum concentrations of sRANKL, OPG or their ratio in patients with JIA compared to healthy control participants. Although studies in RA most often evidence increased sRANKL concentrations [47-49], unchanged concentrations have also been established [50]. In children with JIA, a decrease in RANKL and an increase in OPG have been demonstrated [51]. In other studies of various JIA subtypes, researchers have mentioned an increase in serum as well as synovial fluid sRANKL [15,52-54]. In the present study, sRANKL values of zero were included in the analyses, unlike in some other studies [52,54]. Also, published data about OPG serum concentrations in JIA differ to a high degree, whether they are unchanged [53], increased [51,52] or decreased [15,54]. After TNFα blocker treatment, we did not observe any deviation from baseline sRANKL levels, OPG levels or their ratio. In other studies, investigators have mentioned both sRANKL increases and decreases during treatment with TNFα blockers [55,56]. Similarly, researchers in other studies have described no changes in OPG concentrations during treatment with infliximab or etanercept [55,56].

The advantage of the present study is the prospective follow-up of an age-homogeneous group of both patients and healthy control individuals of both sexes and the testing of a range of variables that describe the quantity, remodelling and regulation of bone mass. The study is limited by the low number of patients with highly heterogeneous therapy, the use of only a single method of sclerostin serum concentration measurement and a lack of information about the source of sclerostin. Further study of local subchondral bone and cartilage expression of sclerostin could yield more valuable information on the pathogenic role of sclerostin in JIA. Incomplete assessment of potential confounding variables, including physical activity, also needs to be taken into account.

## Conclusion

To our knowledge, this study is the first to establish a link between sclerostin serum concentration and the number of tender and swollen joints in patients with JIA. Furthermore, these data support the hypothesis that chondrocytes and cells of the subchondral bone may contribute to circulating sclerostin.

## Abbreviations

25(OH)D: 25-Hydroxyvitamin D_3_
BMD: Bone mineral density
DAS28: Disease Activity Score in 28 joints
Dkk1: Dickkopf Wnt signalling pathway inhibitor 1
ESR: Erythrocyte sedimentation rate
hsCRP: High-sensitivity C-reactive protein
JIA: Juvenile idiopathic arthritis
OC: Osteocalcin
OPG: Osteoprotegerin
PGH: Patient global health
PINP: Procollagen type I N-terminal propeptide
PTH: Parathyroid hormone
sRANKL: Soluble receptor activator of nuclear factor κB ligand
TNFα: Tumour necrosis factor α
βCTX-I: C-terminal telopeptide of collagen type I
